# A Mushroom Extract Piwep from *Phellinus igniarius* Ameliorates Experimental Autoimmune Encephalomyelitis by Inhibiting Immune Cell Infiltration in the Spinal Cord

**DOI:** 10.1155/2014/218274

**Published:** 2014-01-27

**Authors:** Lan Li, Guang Wu, Bo Young Choi, Bong Geom Jang, Jin Hee Kim, Gi Ho Sung, Jae Youl Cho, Sang Won Suh, Hyoung Jin Park

**Affiliations:** ^1^Institute of Medical Science, Hallym University, Chuncheon 200-702, Republic of Korea; ^2^Department of Physiology, College of Medicine, Hallym University, 1-Okcheon-dong, 39 Hallymdaehak-gil, Chuncheon 200-702, Republic of Korea; ^3^Mushroom Research Division, Department of Herbal Crop Research, National Institute of Horticultural & Herbal Science, RDA, Suwon 441-707, Republic of Korea; ^4^Department of Genetic Engineering, Sungkyunkwan University, Suwon 440-746, Republic of Korea

## Abstract

The present study aimed to evaluate the therapeutic potential of a mushroom extract from *Phellinus igniarius* in an animal model of multiple sclerosis. The medicinal mushroom, *Phellinus igniarius*, contains biologically active compounds that modulate the human immune system. Experimental autoimmune encephalomyelitis (EAE) was induced by immunization with myelin oligodendrocyte glycoprotein (MOG 35–55) in C57BL/6 female mice. A water-ethanol extract of *Phellinus igniarius* (Piwep) was delivered intraperitoneally every other day for the entire experimental course. Three weeks after the initial immunization, demyelination and immune cell infiltration in the spinal cord were examined. Piwep injection profoundly decreased the daily incidence rate and clinical score of EAE. The Piwep-mediated inhibition of the clinical course of EAE was accompanied by suppression of demyelination and infiltration of encephalitogenic immune cells including CD4+ T cells, CD8+ T cells, macrophages, and B cells in the spinal cord. Piwep reduced expression of vascular cell adhesion molecule-1 (VCAM-1) in the spinal cord and integrin-*α*_4_ in the lymph node of EAE mice. Piwep also inhibited proliferation of lymphocytes and secretion of interferon-*γ* in the lymph node of EAE mice. The results suggest that a mushroom extract, Piwep, may have a high therapeutic potential for ameliorating multiple sclerosis progression.

## 1. Introduction

Multiple sclerosis (MS) is an autoimmune disease of the central nervous system (CNS) that is associated with inflammation in the CNS and destruction of the myelin sheath [1, 2]. The causes of MS are unknown, but an interplay of genetic factors [3], environmental factors [4], and viral infections [5] have been suggested. However, there are no clear explanations for MS pathology. Although MS afflicts more than one million individuals worldwide and is one of the most common causes of neurological disability in young adults, effective drugs have not yet emerged for its treatment [6].

Since MS is accompanied by both cellular and humoral immunity, immunotherapies with distinct strategies have been attempted [7]. Desensitization of autoreactive T cells with tolerogenic forms of antigen has been previously employed [8]. Monoclonal antibodies against adhesion molecules, T cell tropic cytokines, and B cell surface molecules have also been developed [9]. Plasmapheresis has been also attempted [10]. Although these therapeutic trials exhibit positive results, their use has been limited because of undesirable side effects. Therefore, a medicine that is highly effective and safe is still waiting to be developed for multiple sclerosis patients.

Autoreactive CD4+ T cells that have infiltrated the CNS are reactivated by major histocompatibility complex (MHC) class II molecules presented by antigen presenting cells (APC), proliferate, and differentiate into several subsets that mediate the cell-mediated immunity [11]. CD4+ T cells in the CNS also play a pivotal role in recruitment of other immune cells during development of EAE [12]. Autoreactive CD8+ T cells, once reactivated by MHC class I molecules in the CNS, expand clonally and cytotoxically attack epitope-bearing cells [13]. Macrophages, together with resident microglia in the CNS, recruit other immune cells [14] and activate them [15]. Macrophages secrete proinflammatory cytokines that destroy myelin [16] and phagocytize myelin marked with antibodies and complements [17]. Antigen-specific B cells may function as APC to T cells after infiltration in the CNS [18]. B cells also interact with T cells, which results in simultaneous expansion of antigen-specific B cells and T cells [19]. B cells in the CNS differentiate to plasma cells that secrete antibodies and complements against myelin [17]. Taking these results together along with previous studies, it can be inferred that Piwep may ameliorate EAE by simultaneous suppression of the EAE-associated infiltration of encephalitogenic cells including CD4+ T cells, CD8+ T cells, macrophages, and B cells in the CNS.

Mushrooms belonging to the genus *Phellinus* of the Hymenochaetaceae Basidiomycetes have been traditionally used as a medicine in Asia to treat inflammation and cancers, and their medicinal functions are currently being examined [20]. The most outstanding finding of the medicinal function of the mushroom is that polysaccharides extracted from *Phellinus linteus* have a potent immune-modulating property, which is useful in the treatment of inflammation and cancer [21]. *Phellinus igniarius*, another mushroom belonging to the genus *Phellinus,* has been also used as an herbal medicine in Asia [20] and its immunoregulatory properties have been reported [22]. Polysaccharides, especially *β*-glucan, are believed to be responsible for the biological activity observed in these medicinal mushrooms [23]. Thus, in this study, we aimed to identify potential therapeutic effects of an extract of *Phellinus igniarius* on multiple sclerosis.

One of the most commonly used animal models of multiple sclerosis is experimental autoimmune encephalomyelitis (EAE), which is induced by immunization with myelin oligodendrocyte glycoprotein (MOG) [24]. Using the MOG-induced EAE mouse model, we investigated if systemically delivered mushroom extract (Piwep) could suppress clinical progression and pathological changes. In the present study, Piwep ameliorated the severity of the EAE in MOG-injected mice, which was accompanied with reduction of demyelination, attenuation of microglia activation, and inhibition of infiltrating encephalitogenic immune cells. These results establish that the mushroom extract Piwep has potential therapeutic effects for MS.

## 2. Materials and Methods

### 2.1. Induction of EAE

Animal use and relevant experimental procedures were approved by the Institutional Animal Care and Use Committee, Hallym University (Protocol number Hallym 2011-68). This study was conducted in accordance with the ARRIVE (Animal Research: Reporting In Vivo Experiments) guidelines [25]. C57BL/6 female mice, aged 8 weeks, were purchased from DBL (Chungcheongbuk, Korea), were housed in a temperature- and humidity-controlled environment, and supplied with Purina diet (Purina, Gyeonggi, Korea) and water *ad libitum*. Mice were immunized on day 0 by subcutaneous injection of 200 *μ*L of a mixture of recombinant myelin oligodendrocyte glycoprotein (MOG (35–55), 2 mg/mL) (AnaSpec, CA) and complete Freund's adjuvant containing 400 *μ*g of *Mycobacterium tuberculosis* H37RaA (Difco Laboratories, MI) according to the manufacturer's instruction [26]. Pertussis toxin (List Biological Laboratories, CA) was intraperitoneally administered at a dose of 400 ng on postimmunization days 0 and 2. A booster injection was given on day 7 after the initial immunization.

### 2.2. Preparation of Water-Ethanol Extract of *Phellinus igniarius* (Piwep)

The crude powder of dried fruit bodies of *Phellinus igniarius* (Linnearus: Fries) Quélet 1886 (Amazing Grace Health Product, Bangkok, Thailand) was boiled at 100°C in distilled water for 3 h. After concentration with a rotary evaporator, the water-ethanol extract was mixed with two volumes of cold (−20°C) 95% ethanol and then kept in a refrigerator overnight. The dark brown precipitate was collected after centrifugation. The precipitate was rinsed with 95% ethanol once and suspended in distilled water for freeze-drying. The final product was referred to as a water-ethanol extract of *Phellinus igniarius* (Piwep).

### 2.3. Piwep Treatment of EAE

Piwep was dissolved with normal saline and intraperitoneally injected every other day at a dose of 100 mg/kg/day from day 0 until the end of the experiment. In our preliminary studies, it was observed that the effect of Piwep on EAE was dose-dependent and the optimal dose was 100 mg/kg/day (data not shown).

### 2.4. Behavioral Testing

Behavior was scored daily for evaluation of clinical features of EAE according to the following criteria: 0, no deficit; 0.5, partial loss of tail tone or slightly abnormal gait; 1.0, complete tail paralysis or both partial loss of tail tone and mild hind limb weakness; 1.5, complete tail paralysis and mild hind limb weakness; 2.0, tail paralysis with moderate hind limb weakness (evidenced by frequent foot dropping between bars of cage top while walking); 2.5, no weight-bearing on hind limbs (dragging) but with some leg movement; 3.0, complete hind limb paralysis with no residual movement; 3.5, hind limb paralysis with mild weakness in forelimbs; 4.0, complete quadriplegia but with some movement of head; 4.5, moribund; 5, dead [27].

### 2.5. Pathological Examination of the Central Nervous System

On day 21 after the initial immunization, mice were transcardially perfused with 4% paraformaldehyde in phosphate-buffered saline (PBS) under an anesthesia with Zoletil 50 and Xylazine. The spinal cord and brain were removed and postfixed in the same fixative. After embedding the spinal cord in paraffin, sections at 5 *μ*m were made and then stained with Luxol Fast Blue (LFB) and Periodic Acid Schiff (PAS) to determine demyelination. Frozen sections at 30 *μ*m were stained with Cresyl violet to detect inflammatory cell infiltration.

### 2.6. Immunohistochemical Examination of the Spinal Cord and the Lymph Node

The spinal cord was obtained from mice previously perfused with 4% paraformaldehyde. The spinal cord was postfixed in the same fixative and immersed in PBS containing 30% sucrose at 4°C for 2 days. Frozen sections at 30 *μ*m were immunohistochemically stained with specific antibodies against cell surface molecules for cluster differentiation (CD) according to the conventional method. Monoclonal antibodies against CD4 (BD Bioscience, San Jose, CA, USA), CD8 (BD Bioscience), F4/80 (eBioscience, San Jose, CA, USA), or a polyclonal antibody against CD20 produced in goat (SantaCruz Biotechnology, Santa Cruz, CA, USA) were used as the primary antibody. An antibody against rat IgG or goat IgG (Vector Laboratories, Burlingame, CA, USA) was employed as the secondary antibody. Cervical lymph nodes were fixed in the neutral formaldehyde solution for 2 days. After embedding the lymph node in paraffin, sections at 5 *μ*m were made and then immunohistochemically stained with specific monoclonal antibody against integrin-*α*_4_ (Merck, Whitehouse Station, NJ). An antibody against mouse IgG (Vector Laboratories, Burlingame, CA) was employed as the secondary antibody. The immunoreaction was visualized with 0.06% 3, 3′-diaminobenzidine (Dako, Denmark) in 0.1 M Tris buffer after incubation in ABC reagent (Vector Laboratories, Burlingame, CA).

### 2.7. Reverse Transcription Polymerase Chain Reaction (RT-PCR) Analysis of mRNA in the Spinal Cord and Lymph Nodes

The spinal cord and lymph nodes were obtained from mice after being perfused with cold PBS. Extraction of total RNA was performed with the Trizol reagent (Invitrogen, Camarillo, CA, USA). RT-PCR was performed to measure the levels of mRNA specific for CD4, CD8, CD11b, CD20, vascular cell adhesion molecule-1, and integrin-*α*_4_ in the extract according to the manufacturer's instructions. First-strand cDNA was synthesized from 1 *μ*g of total RNA using the cDNA synthesis kit (TaKaRa Bio, Shiga, Japan) according to the manufacturer's instruction. The oligonucleotide primers were as follows: CD4 forward 5′-TGT GCC GAG CCA TCT CTC TTA GG-3′, reverse 5′-GCA CTG AGA GTG TCA TGC CGA AC-3′; CD8 forward 5′-ATG CAG CCA TGG CTC TGG C-3′, reverse 5′-GCA TGT CAG GCC CTT CTG GGT-3′; CD11b forward 5′-GGG CAC GGT GGC AGG TGA A-3′, reverse 5′-GCT GGC TGT GGG AGG CAC TG-3′; CD20 forward 5′-AAA ACC TCC AGG AAG AGT TTG GTC-3′, reverse 5′-CGA TCT CAT TTT CCA CTG GCA AG-3′; vascular cell adhesion molecule (VCAM)-1 forward 5′-AGG CAC AGC TGC AGG ATG CC-3′, reverse 5′-GGA GGG GGC GGG GCT GTA AT-3′; integrin-*α*_4_ forward 5′-CCA CTA CGA TCG CTC CGC CTG T-3′, reverse 5′-CCA CTA CGA TCG CTC CGC CTG T-3′, *β*-Actin forward 5′-TGG AAT CCT GTG GCA TCC ATG AAA C-3′, reverse 5′-TAA AAC GCA GCT CAG TAA CAG TCC G-3′. RT-PCR products were separated on an agarose gel, and then RNA bands were quantified using Image J (NCBI, Bethesda, MD, USA) after staining with ethidium bromide.

### 2.8. Proliferation and Cytokine Production of Lymphocytes in the Regional Lymph Node

On day 21 after the 1st immunization, lymphocytes were prepared from regional lymph nodes of the MOG-immunized mice treated with or without Piwep. Briefly, lymphocytes were released by teasing into RPMI1640 medium (Lonza, Basel, Switzerland) supplemented with 20 mM HEPES buffer (Gibco, NY). Lymphocytes were washed three times in Ca^2+^, Mg^2+^-free Hank's balanced salt solution (PAA Laboratories, Linz, Austria) and resuspended to 5 × 10^6^ cells/mL in RPMI1640 medium containing 100 U/mL of penicillin, 100 mg/mL of streptomycin, and 10% fetal bovine serum (PAA Laboratories). Lymphocytes (5 × 10^6^ cells/mL) were cultured in 96-well plates in the presence of MOG (10 *μ*g/mL) in a total volume of 200 *μ*L/well for 48 h. Cell proliferation was measured by the conventional 3-(4,5-dimethylthiazol-2-yl)-2,5-diphenyltetrazolium bromide (MTT; Sigma, MO) assay. At 4 h prior to termination of culture, 10 *μ*L of MTT solution (10 mg/mL of MTT in PBS) was added in the culture medium. Cultures were lysed by addition of 15% sodium dodecyl sulfate (SDS) into each well for solubilization of formazan and the optical density at 570 nm was measured by SpectraMax 250 microplate reader (BioTek, Winooski, VT, USA). Concentrations of IL-12 and IFN-*γ* in the culture media were determined with ELISA kits (Amersham, Little Chalfont, Buckinghamshire, UK) after incubation of lymphocytes (5 × 10^6^ cells/mL) in the presence of MOG (10 *μ*g/mL) for 48 h.

### 2.9. Statistical Analysis

EAE clinical scores were reported as average ± SEM. Repeated measure analysis of variance (ANOVA) was used for statistical analysis of these data. The fluorescence intensity and T cell proliferation data were expressed as the mean ± SEM and analyzed for statistical significance using one-way ANOVA, followed by a Bonferroni post hoc test. *P* < 0.05 was considered to be significant.

## 3. Results 

### 3.1. Piwep Ameliorates Clinical Signs of MOG-Induced EAE and Disease Progression

**Figure 1:**
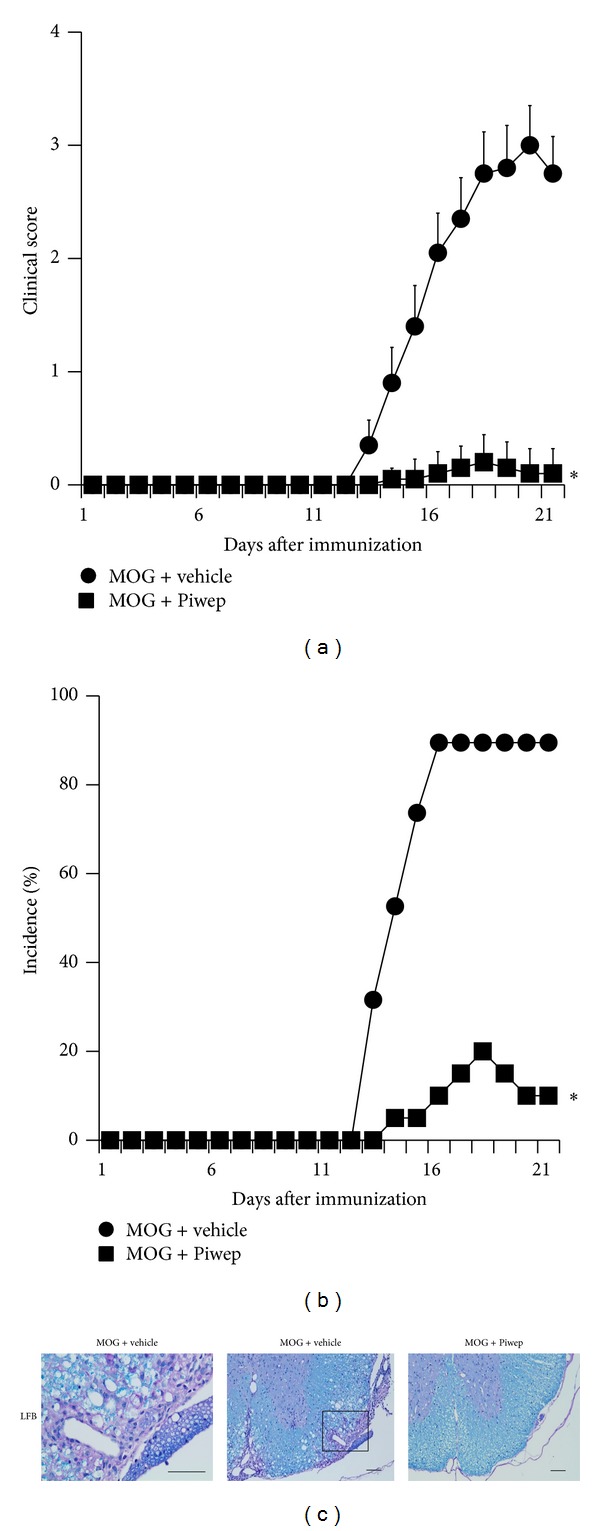
Piwep ameliorates the clinical signs and disease progression of myelin oligodendrocyte glycoprotein (MOG) induced EAE. Clinical scores were recorded daily until 21 days after EAE induction. The clinical signs of EAE first appeared on day 13 and reached peak levels on day 20 from the 1st MOG (M) immunization. Piwep (MOG + Piwep) or its vehicle (MOG + vehicle) was intraperitoneally administered during the entire period. (a) Piwep profoundly reduced the EAE clinical score induced by MOG. Data are mean ± SEM (*n* = 15 each group) *P < 0.05 compared with Piwep treated group. (b) Piwep reduced the incidence rate of EAE induced by MOG. (c) Piwep reduced MOG-induced demyelination in spinal cord of EAE mice. On day 21 after the initial immunization, demyelination in the spinal cord was visualized with LFB staining. White matter damage is reflected by reduced LFB staining in the spinal cord. (c) LFB staining of the spinal cord showed extensive demyelination in the MOG-immunized EAE mice. EAE-associated demyelination is almost completely absent in EAE mice treated with Piwep. The square-shaped area in the low power magnification shown in the left panel was enlarged four hundred times and illustrated in the right panel. Scale bar represents 100 *μ*m.

When EAE was induced in mice with recombinant MOG (35–55), clinical signs of EAE first appeared on day 13 and reached a peak level of 3.00 ± 0.40 on day 20 after the initial immunization. Mice immunized with MOG (35–55) developed severe EAE symptoms with complete hind limb paralysis (EAE incidence rate 21/21). The daily clinical course of EAE was inhibited by Piwep as shown in Figures 1(a) and 1(b). The maximal clinical score of EAE was significantly (*P* < 0.001) lower in the MOG + Piwep group (0.20 ± 0.09) than that in the MOG + vehicle group (3.15 ± 0.39). The incidence rate of EAE was also much lower in the MOG + Piwep group (20%, 4 of 20) than that in the MOG + Vehicle group (90%, 18 of 20). LFB & PAS staining of the spinal cord exhibited that EAE was accompanied by severe demyelination in the white mater. Compared with the vehicle-treated EAE group, the Piwep-treated group markedly diminished the demyelination (Figure 1(c)).

### 3.2. Piwep Treatment Attenuates EAE-Induced Mononuclear Cell Infiltration into the White Matter of Spinal Cord and Cerebellum in Mice

**Figure 2:**
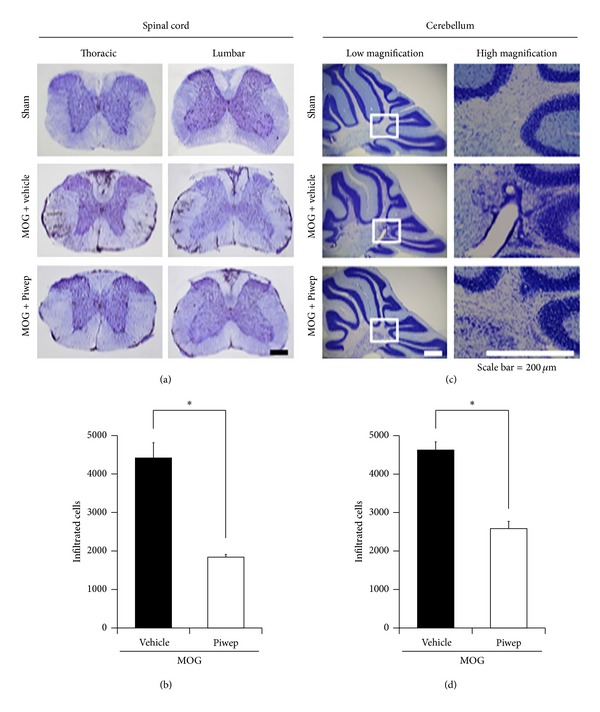
Piwep treatment attenuates EAE-induced mononuclear cell infiltration into the white matter of spinal cord, cerebral cortex, and cerebellum in mice. Three weeks after the initial immunization of MOG, infiltration of mononuclear cells around small vessels in the spinal cord and cerebellum was detected with cresyl violet staining. EAE mice revealed intensive infiltration of mononuclear cells around the white matter of spinal cord (a, b) and cerebellum (c, d). However, Piwep treatment reduced mononuclear cell infiltration into the white matter of the spinal cord and cerebellum. Scale bar represents 100 *μ*m. Data are mean ± SEM (*n* = 3). *P < 0.05 compared with Piwep treated group.

Three weeks after the initial immunization of MOG, infiltration of mononuclear cells around small vessels in the spinal cord and cerebellum was detected with cresyl violet staining. EAE mice revealed intensive infiltration of mononuclear cells around the white matter of spinal cord and cerebellum. However, Piwep treatment reduced mononuclear cell infiltration into the white matter of the spinal cord and cerebellum (Figure 2).

### 3.3. Piwep Suppresses Immune Cell Infiltration in the Spinal Cord of EAE Mice

**Figure 3:**
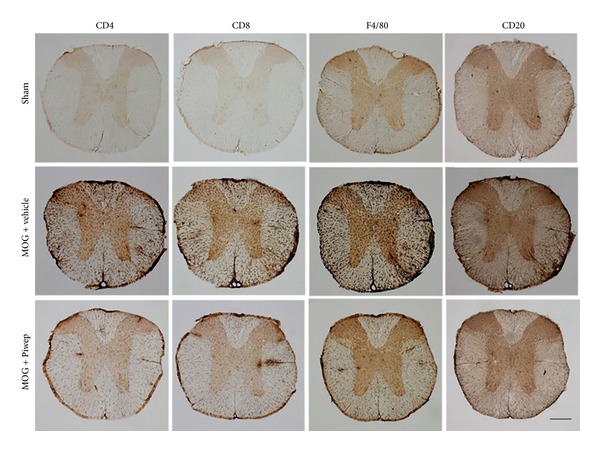
Piwep treatment attenuates EAE-induced immune cell infiltration into white matter of spinal cord in mice. PFA fixed sections of the thoracic spinal cord were immunohistochemically stained with antibodies against cell surface molecules such as CD4, CD8, F4/80, and CD20. Immunostaining revealed that T cell, B cell, and microglia/macrophage labeled cells extensively infiltrated the white matter of EAE mice (MOG + vehicle). However, Piwep treatment reduced the infiltration of immune cells into the white matter of the spinal cords in EAE mice (MOG + Piwep). Scale bar represents 100 *μ*m.

**Figure 4:**
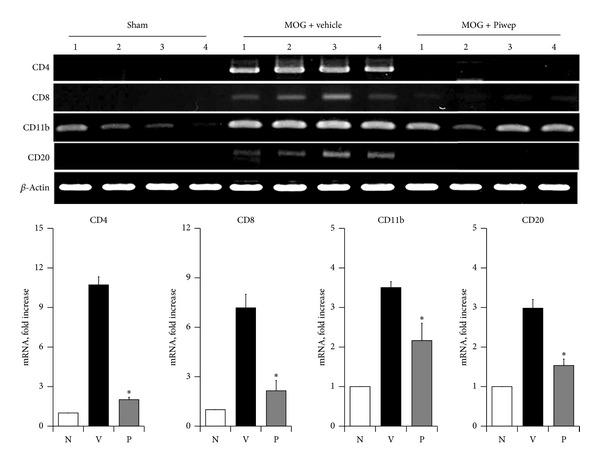
Piwep treatment attenuates mRNA expression of surface molecules in the spinal cord of EAE mice. Spinal cord perfused with cold PBS was used for extraction of total RNA. Expression of mRNA was determined with the RT-PCR method. RT-PCR results demonstrate that T cell, B cell, and microglia/macrophage labeled cells extensively infiltrated the white matter of EAE mice (MOG + vehicle). However, Piwep significantly reduced the EAE-associated increase in mRNA expression of CD11b, CD4, CD8, and CD20. Graphs represent quantified CD11b, CD4, CD8, and CD20 expression with or without Piwep treatment in EAE mice. Data are mean ± SEM (*n* = 4). *P < 0.05 compared with Piwep treated group.

The spinal cord was examined to identify subpopulations of immune cells affected by Piwep.  Figure 3 shows immunohistochemical staining of the spinal cord with antibodies specific for cell surface molecules. Immunoreactivity of CD4, CD8, F4/80, and CD20 was increased in the spinal cord with EAE, all of which were markedly reduced by Piwep.  Figure 4 demonstrates mRNA expression of cell surface molecules in the spinal cord. RT-PCR analysis of cell surface molecules exhibited that mRNA expression of CD4, CD8, CD11b, and CD20 was upregulated in the spinal cord of EAE mice. Piwep also significantly (*P* < 0.05) inhibited the EAE-associated increase in mRNA expression of these cell surface molecules.

### 3.4. Piwep Suppresses Expression of VCAM-1 and Integrin-*α*_4_ mRNA in the Spinal Cord of EAE Mice

**Figure 5:**
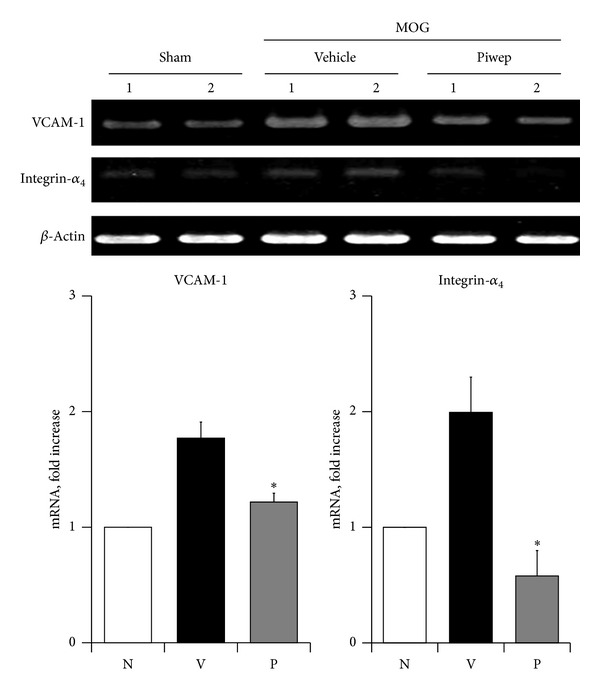
Piwep treatment attenuates mRNA expression of vascular cell adhesion molecule in the spinal cord of EAE mice. Spinal cord perfused with cold PBS was used for extraction of total RNA. Expression of mRNA was determined with the RT-PCR method. RT-PCR results demonstrated an increase of vascular cell adhesion molecule-1 (VCAM-1) and its ligand, integrin-*α*_4_ in MOG-induced EAE mice spinal cord. Piwep significantly inhibited VCAM-1 and integrin-*α*_4_ expression in the spinal cord. Data are mean ± SEM (*n* = 2). *P < 0.05 compared with Piwep treated group.

Expression of mRNA coding for cell adhesion molecules was analyzed because they are necessary for transmigration of lymphocytes through the vessels in the spinal cord. As illustrated in Figure 5, RT-PCR analysis exhibited that mRNA of vascular cell adhesion molecule (VCAM)-1 and integrin-*α*_4_ were highly upregulated in the spinal cord of EAE mice. Piwep significantly (*P* < 0.05) inhibited the EAE-associated increase in mRNA expression of these adhesion molecules.

### 3.5. Piwep Inhibits Lymphocyte Activity in the Regional Lymph Node of EAE Mice

**Figure 6:**
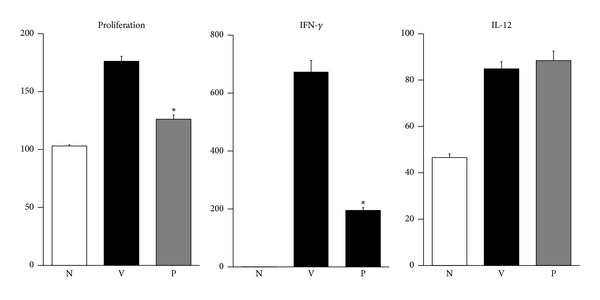
Piwep inhibits lymphocyte activity in the regional lymph node of EAE mice. Effects of Piwep on lymphocyte activity in the regional lymph node—where lymphocytes proliferate—were determined. EAE was associated with not only proliferation but also with secretion of interferon (IFN)-*γ* and interleukin (IL)-12 of lymphocytes obtained from cervical lymph nodes. Piwep significantly (*P* < 0.05) inhibited the EAE-associated proliferation and interferon-*γ* secretion, except IL-12. Data are mean ± SEM (*n* = 5). *P < 0.05 compared with Piwep treated group.

Effects of Piwep on the lymphocyte activity in the regional lymph node were determined because the lymph node is the primary site of lymphocyte proliferation. As shown in Figure 6, EAE was associated with not only proliferation but also secretion of interferon (IFN)-*γ* and interleukin (IL)-12 of lymphocytes obtained from cervical lymph nodes [28, 29]. Piwep significantly (*P* < 0.05) inhibited the EAE-associated proliferation and interferon-*γ* secretion, except IL-12.

### 3.6. Piwep Reduces Expression of Integrin-*α*_4_ in the Cervical Lymph Node of EAE Mice

**Figure 7:**
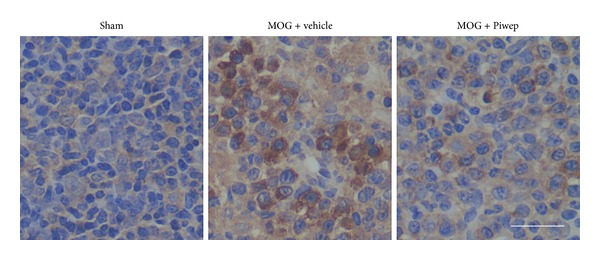
Piwep reduces expression of integrin-*α*_4_ in the cervical lymph node of EAE mice. Integrin-*α*_4_, a cell adhesion molecule, is highly expressed on lymphocytes that infiltrate the central nervous system. Immunohistochemical staining revealed that the number of lymphocytes with high expression of integrin-*α*_4_ increased in cervical lymph nodes 21 days after the 1st immunization. Piwep reduced the EAE-induced increase in number of lymphocytes immunoreactive to integrin-*α*_4_ in cervical lymph nodes. Scale bar represents 100 *μ*m.

Integrin-*α*_4_, a cell adhesion molecule, should be highly expressed on lymphocytes that infiltrate the central nervous system. As demonstrated in Figure 7, immunohistochemical staining revealed that number of lymphocytes with high expression of integrin-*α*_4_ in cervical lymph nodes 21 days after the 1st immunization. Piwep reduced the EAE-induced increase in number of lymphocytes immunoreactive to integrin-*α*_4_ in cervical lymph nodes.

## 4. Discussion

In the present study, Piwep profoundly inhibited the incidence and clinical score of experimental autoimmune encephalomyelitis (EAE). It is well known that inflammation and demyelination in the central nervous system (CNS) are hallmarks of neuropathological changes that accompany EAE [30]. Thus, the effects of Piwep on the neuropathological changes were investigated in this study. EAE was accompanied by intensive infiltration of mononuclear cells around blood vessels and severe demyelination in the white matter. The neuropathological changes associated with EAE were hardly observed when EAE mice were treated with Piwep, a mushroom extract from *Phellinus igniarius*. These results clearly demonstrate that Piwep inhibits the clinical features and neuropathological changes of EAE.

Because the EAE-associated infiltration of mononuclear cells was modified by Piwep, the subpopulation of immune cells infiltrating the spinal cord was analyzed in this study. Immunohistochemistry of the spinal cord with antibodies specific for cell surface molecules for cluster differentiation (CD) revealed that immunoreactivities of CD4, CD8, F4/80, and CD20 remarkably increased in the EAE mouse. Piwep markedly inhibited the increase in immunoreactivities for these antigens. Subpopulations of immune cells in the spinal cord were also analyzed by quantification of mRNA expression of cell surface molecules. RT-PCR studies also exhibited that expression of CD4, CD8, CD11b, and CD20 mRNA was coincidentally upregulated in the spinal cord of EAE mice. Piwep also remarkably inhibited the EAE-associated increase in mRNA expression of these molecules. The results strongly indicate that Piwep may inhibit migration of all subpopulations of immune cells into the CNS.

Most of the beneficial effects of medicinal mushroom are concentrated on its immune modulating effects. It has been documented that medicinal mushroom differentially activated the immune system when challenged by a variety of extracellular stimuli. Medicinal mushroom has been shown to modulate diverse systemic responses to help restore a balanced immune response [21, 31–33]. Administration of proteoglycans derived from medicinal mushroom also prevented collagen-induced arthritis (CIA) in mice as an experimental model of autoimmune disease [34].

Endothelial vascular cell adhesion molecules are considered as key mediators of leukocyte recruitment. Vascular cell adhesion molecule-1 (VCAM-1) has been shown to be important in the development of experimental autoimmune encephalomyelitis (EAE) and MS, mediating both leukocyte movements across the BBB and their retention within the parenchyma [35]. VCAM-1 expression occurs in advance of BBB breakdown, allowing leukocytes to cross the BBB. Previous studies have suggested that increased BBB permeability precedes the occurrence of histological lesions [36]. Selective blockade of the interaction between VCAM-1 and its ligand, integrin-*α*_4_, on leukocytes has been shown to abolish leukocyte recruitment and the associated neurological deficit in EAE models [37]. Similarly, integrin-*α*_4_ inhibitors reduce the number of lesions in MS [38]. Our present study shows that Piwep significantly inhibited VCAM-1 expression in the spinal cord. However, how Piwep inhibits the VCAM-1 expression in the spinal cord should be further investigated in subsequent studies.

It is also unknown at the present time which substance(s) in Piwep exerts the inhibitory effect on EAE. Various bioactive substances including polysaccharides, cyclophellitol, furan derivatives, hispidin, and hispolon have been identified from extract of *Phellinus linteus*, another mushroom belonging to the genus *Phellinus* [21]. Several therapeutic polysaccharides such as hetero-*β*-glucans and their protein complexes (e.g., xyloglucans and acidic *β*-glucan-containing uronic acid) have been isolated from medicinal mushrooms [23]. The active component(s) in Piwep should be elucidated in future studies.

In summary, a mushroom extract Piwep ameliorates EAE-induced clinical course in mice. The amelioration was accompanied by inhibition of the EAE-associated demyelination and infiltration of encephalitogenic immune cells including CD4+ T cells, CD8+ T cells, macrophages, and B cells in the CNS. Therefore, taken together, our results suggest that a very potent and safe therapeutic agent for MS patients could be developed from the polysaccharide-enriched fraction obtained from *Phellinus igniarius*.
